# Afibrinogénémie congénitale: à propos d’une observation

**DOI:** 10.11604/pamj.2016.25.233.10754

**Published:** 2016-12-09

**Authors:** Karim Assani, Lamya Karboubi, Badr Sououd Benjelloun Dakhama

**Affiliations:** 1 Service des Urgences Pédiatriques, Hôpital d’Enfants, Rabat, CHU Ibn-Sina, Maroc

**Keywords:** Afibrinogénémie, troubles de coagulation, concentré de fibrinogène, Afibrinogenemia, blood clotting disorders, fibrinogen concentrate

## Abstract

L’afibrinogénémie est une dyscrasie rare caractérisée par un déficit constitutionnel en fibrinogène. Elle se transmet sur un mode autosomique récessif. Les manifestations hémorragiques sont variables et peuvent mettre en jeu le pronostic vital. Un peu plus de 250 cas ont été publiés à nos jours. Nous rapportons un nouveau cas d’afibrinogénémie congénitale chez une enfant de 3 ans et demi hospitalisée pour hématémèse de moyenne abondance. A travers ce cas, nous rappelons les différents aspects de cette affection sur les plans clinique, biologique, génétique ainsi que thérapeutique.

## Abstract (en)

Afibrinogenemia is a rare dyscrasia characterized by a congenital fibrinogen deficiency It is transmitted in an autosomal recessive manner. Hemorrhagic manifestations are variable and can be life-threatening. A little more than 250 cases have been published up till now. We here report a new case of congenital afibrinogenemia in a 3 1/2-year old child hospitalized for medium abundance hematemesis. This case study aims to highlight numerous aspects of this condition from a clinical, biological, genetic and therapeutic points of view.

## Introduction

L’afibrinogénémie congénitale est une maladie héréditaire très rare. Sa prévalence est estimée à 1/1000 000 et sa transmission se fait selon le mode autosomique récessif [1]. Environ 150 familles ont été rapportées à travers le monde depuis la première observation publiée par deux médecins allemands Rabe et Salomon en 1920. Cette maladie est due à un déficit constitutionnel en fibrinogène, glycoprotéine plasmatique synthétisée par l’hépatocyte. Elle peut se manifester en période néonatale par une hémorragie du cordon ou plus tard par des manifestations hémorragiques dont certaines pouvant mettre en jeu le pronostic vital. Nous rapportons un cas d’afibrinogénémie congénitale révélée par des saignements récurrents.

## Patient et observation

Fille de 3 ans et demi, unique de sa famille, était admise pour hématémèse de moyenne abondance. Elle présentait dans ses antécédents une notion d’hémorragie du cordon ombilicale en période néonatale, des ecchymoses au moindre traumatisme, des épisodes de gingivorragies et d’épistaxis de faible abondance dont un a nécessité une hospitalisation à l’âge de 2 ans et demie. Il n’y a pas de cas similaire dans la famille. A noter que les parents sont consanguins au premier degré. A son admission, la patiente était en assez bon état général, un état hémodynamique stable, apyrétique avec une pâleur cutanéomuqueuse. L’abdomen était souple, sans masse palpable, sans splénomégalie ni hépatomégalie. L’auscultation cardiopulmonaire était normale. Le reste de l’examen clinique était sans particularité. Le bilan montrait une anémie à 8 g/dl, hypochrome microcytaire avec une ferritinémie basse à 7 ng/ml, une leucocytose normale à 7300 éléments/mm3 et un taux de plaquettes également normal à 240 000 éléments/mm3. Le bilan de crase montrait un temps de céphaline activé (TCA) allongé à 60 secondes ainsi qu’un temps de Quick allongé avec un temps de prothrombine (TP) à 45%, le fibrinogène indétectable au dosage, et une absence des produits de dégradation de la fibrine (PDF). Parallèlement, les facteurs de la coagulation II, VII, VIII, IX et X étaient normaux. A noter que les parents sont asymptomatiques et leurs bilans biologiques se sont avérés normaux. Le diagnostic d’afibrinogénémie congénitale a été retenu. L’évolution clinique durant son admission a été marquée par la répétition des hématémèses nécessitant une prise en charge en réanimation pour transfusion et surveillance. L’enfant a reçu un traitement substitutif à base de plasma frais congelé avec bonne évolution.

## Discussion

L’afibrinogénémie est une affection très rare. Sa fréquence est estimée au niveau international à 1-2 cas par million de personnes [1]. Contrairement à l’hémophilie A où la prévalence est stable autour de 1/5000 sujets de sexe masculin, l’incidence de cas d’afibrinogénémie à la naissance varie suivant les régions. Cette variation est essentiellement expliquée par l’endogamie. Ainsi, dans les populations où la consanguinité est fréquente comme il a été noté dans le registre iranien, l’incidence des troubles du fibrinogène est 7 fois plus grande en comparaison avec d’autres registres similaires en Italie et au Royaume-Uni [2–4]. Au Maroc, son incidence reste sous-estimée du fait de la difficulté d’accès aux soins de certaines populations. La transmission se fait selon un mode autosomique récessif et la consanguinité est retrouvée chez 50% des familles, les parents étant asymptomatiques. Le sex ratio est de 1. Au Maroc, deux études ont été faites et les sujets étudiés étaient de sexe féminin [5, 6]. L’âge au diagnostic varie. L’hémorragie du cordon ombilicale est un signe fréquent en période néonatale. Selon une étude japonaise menée sur 14 patients atteints d’afibrinogénémie, ce signe a été retrouvé dans 92.8% des cas et l’âge moyen au diagnostic était de 6 ans [7]. Bien que l’afibrinogénémie soit congénitale, les saignements peuvent survenir tardivement et certains patients sont diagnostiqués seulement vers la deuxième décade de la vie. Cliniquement, les symptômes s’apparentent à ceux de l’hémophilie. En période néonatale, l’hémorragie ombilicale peut être suivie par des hématomes sous cutanées liés à des traumatismes de naissance, rarement une hémorragie du tractus gastro-intestinal. A distance de la période néonatale, il y a une tendance aux saignements de gravité et à intervalles de temps variables : ecchymoses, épistaxis, gingivorragies, hémarthroses, hémorragie gastro-intestinale ou génito-urinaire. Certaines localisations de saignements peuvent menacer le pronostic vital comme c’est le cas de l’hémorragie intracrânienne, thoracique ou abdominale ou menacer le pronostic fonctionnel en cas d’hémorragie intraoculaire [8, 9]. Ces manifestations peuvent survenir spontanément ou suite à un traumatisme. Chez notre patiente, la notion d’hémorragie ombilicale est notée et les ecchymoses à répétitions ont dominé le tableau clinique mais le diagnostic n’a été posé qu’à l’âge de 3 ans et demi. Paradoxalement aux symptômes hémorragiques, des manifestations thromboemboliques peuvent survenir [8, 9]. Une particularité est à noter chez les filles atteintes d’afibrinogénémie sur l’impact de cette maladie sur les menstruations et la conception. Il existe un risque accru de ménorragies pouvant nécessiter une contraception orale et une tendance aux avortements spontanés avec hémorragies post-abortales. Un cas de grossesse menée à terme chez une femme atteinte d’afibrinogénémie a été rapporté [10].

**Figure 1:**
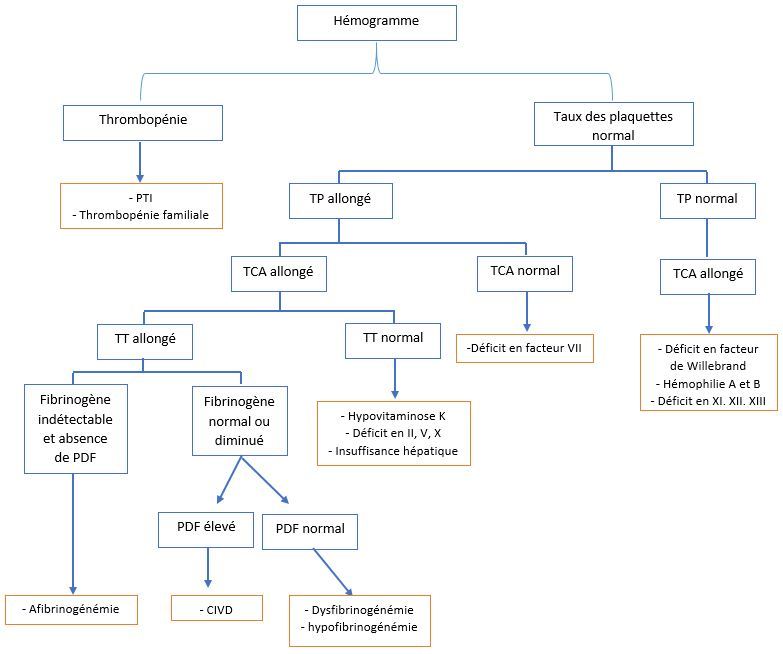
Orientation biologique pour le diagnostic d’un saignement récidivant chez l’enfant (PTI: purpura thrombopénique immunologique, TP: temps de prothrombine, TCA: temps de céphaline activée, TT: temps de thrombine, PDF: produits de dégradation de fibrine, CIVD: coagulation intravasculaire disséminée)

Sur le plan biologique, l’afibrinogénémie est caractérisée par un sang incoagulable avec tous les tests explorant la coagulation globale perturbés: temps céphaline kaolin, temps de Quick, temps de thrombine. Le fibrinogène est indétectable par les méthodes de mesure conventionnelles, chronométrique, pondérale ou immunologique, bien que des traces peuvent être détectées par des méthodes plus sensibles radio-immunologiques ou immuno-enzymologiques [11]. Le taux de plaquettes est normal. Au niveau de l’hémostase primaire, l’afibrinogénémie entraine une réduction de l’agrégation plaquettaire se traduisant par un temps de saignement allongé. Tous les facteurs de coagulation, à l’exception du fibrinogène, présentent des taux normaux. Les produits de dégradation de fibrine sont négatifs [11, 12]. Les diagnostics différentiels en fonction de l’orientation biologique lors d’un saignement récidivant chez l’enfant sont discutés sur la Figure 1. Le fibrinogène est une glycoprotéine formée par trois sous unités (chaines α, β, γ). Ces 3 chaînes polypeptidiques sont synthétisées séparément à partir des gènes situés sur le tiers distal du bras long du chromosome 4 (FGA: fibrinogen alpha chain, FGB: fibrinogen beta chain, FGG: fibrinogen gamma chain) [12, 13]. Cette synthèse se fait essentiellement dans l’hépatocyte. L’afibrinogénémie est due à une anomalie des gènes codant pour le fibrinogène et non pas à une dégradation excessive du fibrinogène ou à un défaut dans la régulation commune à l’expression des trois gènes. Plusieurs mutations ont été identifiées et 80% de ces mutations se situent sur le gène FGA [14–16]. Chez notre patiente, une analyse génétique pour recherche du type de mutation a été demandée mais n’a pas abouti. Le conseil génétique a été fait et la famille a été sensibilisée pour un dépistage en cas de nouvelle naissance. La prise en charge est basée sur la prévention des traumatismes et une thérapeutique de substitution en période hémorragique ainsi qu’en prophylaxie [1, 17]. Il a été observé après transplantation hépatique une résolution de l’afibrinogénémie congénitale [18]. La thérapie génique est une approche thérapeutique d’avenir. La prévention consiste en l’éviction des activités sportives à risque de traumatisme même minime, l’éducation des parents et de l’enfant, l’apprentissage des gestes à faire en cas de saignement, et la nécessité de consulter rapidement en cas des symptômes sévères. Le traitement substitutif a pour but d’élever le taux sanguin du fibrinogène à un niveau permettant d’assurer une hémostase normale [1]. La durée du traitement variera en fonction de l’importance et du type de saignement [1, 19]. Le traitement substitutif à but prophylactique peut être envisagé chez tout patient dès le diagnostic ou plus tard dans la vie chez les patients ayant une forte tendance aux saignements ou particulièrement pendant la grossesse. Les produits utilisés sont essentiellement le plasma frais congelé, le cryoprécipité et les concentrés de fibrinogène. Ces derniers constituent le traitement de choix en raison de leur innocuité, leur efficacité et le faible volume à transfuser mais leur coût limite leur utilisation dans les pays à faible revenu. La thérapeutique de substitution dans le contrôle des épisodes hémorragiques graves et durant la chirurgie vise des taux sanguins de fibrinogène pouvant aller au-delà de 2 g/l [20]. Devant la non disponibilité des concentrés de fibrinogène, notre patiente a reçu le plasma frais congelé.

## Conclusion

L’afibrinogénémie congénitale est une dyscrasie rare. Son diagnostic positif est facile devant un tableau clinique évocateur et les anomalies à la biologie. Malgré une variabilité clinique, il convient d’évoquer ce diagnostic devant un saignement récidivant chez l’enfant et particulièrement devant une hémorragie néonatale. Le diagnostic posé précocement permet une prise en charge adaptée afin d’éviter des hémorragies sévères pouvant mettre en jeu le pronostic vital ou fonctionnel de l’enfant. La prise en charge se conçoit de préférence dans des centres spécialisés et le traitement repose pour l’essentiel à une prophylaxie et l’administration des concentrés de fibrinogène devant un syndrome hémorragique.
